# Parkin- and PINK1-Dependent Mitophagy in Neurons: Will the Real Pathway Please Stand Up?

**DOI:** 10.3389/fneur.2013.00100

**Published:** 2013-07-19

**Authors:** Karl Grenier, Gian-Luca McLelland, Edward A. Fon

**Affiliations:** ^1^McGill Parkinson Program, Department of Neurology and Neurosurgery, Montreal Neurological Institute, McGill University, Montreal, QC, Canada

**Keywords:** autophagy, Mitochondria, mitophagy, neurons, parkin, Parkinson’s disease, PINK1

## Abstract

Parkinson’s disease (PD) is characterized by massive degeneration of dopaminergic neurons in the *substantia nigra*. Whereas the majority of PD cases are sporadic, about 5–10% of cases are familial and associated with genetic factors. The loss of parkin or PINK1, two such factors, leads to an early onset form of PD. Importantly, recent studies have shown that parkin functions downstream of PINK1 in a common genetic pathway affecting mitochondrial homeostasis. More precisely, parkin has been shown to mediate the autophagy of damaged mitochondria (mitophagy) in a PINK1-dependent manner. However, much of the work characterizing this pathway has been carried out in immortalized cell lines overexpressing high levels of parkin. In contrast, whether or how endogenous parkin and PINK1 contribute to mitophagy in neurons is much less clear. Here we review recent work addressing the role of parkin/PINK1-dependent mitophagy in neurons. Clearly, it appears that mitophagy pathways differ spatially and kinetically in neurons and immortalized cells, and therefore might diverge in their ultimate outcome and function. While evidence suggests that parkin can translocate to mitochondria in neurons, the function and mechanism of mitophagy downstream of parkin recruitment in neurons remains to be clarified. Moreover, it is noteworthy that most work has focused on the downstream signaling events in parkin/PINK1 mitophagy, whereas the upstream signaling pathways remain comparatively poorly characterized. Identifying the upstream signaling mechanisms that trigger parkin/PINK1 mitophagy will help to explain the nature of the insults affecting mitochondrial function in PD, and a better understanding of these pathways in neurons will be the key in identifying new therapeutic targets in PD.

## Introduction

Over the past two decades, the identification of genes responsible for complex neurodegenerative disorders has profoundly changed our understanding of pathogenic mechanisms leading to neuronal cell death. Whereas previous research focused on post-mortem studies, analysis of the physiological function of causative genes now allows us to focus directly on key cellular pathways involved in pathology (1).

Among these pathways, dysregulation of mitochondrial quality control has emerged as a common theme for many neurological diseases and in particular for Parkinson’s disease (PD) (2). Mitochondrial dysfunction has been a longstanding theme in PD following observations that mitochondrial toxins such as MPTP and rotenone could induce acute parkinsonism (3, 4) and that mitochondrial respiration was defective in the *substantia nigra pars compacta* (SNpc) of post-mortem PD patients (5). Furthermore, the SNpc of patient brains has been shown to have a higher occurrence of mtDNA deletions compared to aged-matched controls (6, 7).

More recently, two PD-linked genes – parkin and PINK1 – have been implicated in mitochondrial quality control, via the degradation of dysfunctional mitochondria by autophagy (a process termed mitophagy). This suggests that the mitochondrial dysfunction observed in PD may be the result of compromised mitochondrial quality control mechanisms. Most studies examining this process, however, have employed immortalized cell lines in place of primary cell cultures, and few have studied it in neurons. In this review, we summarize the evidence for a physiological role for mitophagy in neurons, discussing the possible role of parkin and PINK1 in such a pathway and its relevance to PD.

## Mitochondrial Dynamics and Quality Control in Health and Disease

Mitochondria – double membrane-bound organelles originating from the symbiosis between an early eukaryotic cell and a prokaryotic cell – are essential for generating energy through the process of oxidative phosphorylation (OXPHOS) and also play important roles in fatty acid metabolism, apoptosis, and calcium-buffering (8).

Long regarded as individual, “bean-shaped” organelles, mitochondria are now understood as a dynamic, inter-connected network, linked to other organelles and important players in a myriad of cellular signaling pathways (9). By regulating the connectivity and the size of the mitochondrial network, the cell can regulate energy production and most other mitochondrial processes (9). While the shape of the mitochondrial network is controlled by fusion- and fission-specific GTPases, the size of the network is controlled by *de novo* mitochondrial biogenesis and macroautophagy.

Mitochondria are the cellular site of OXPHOS, as well as many other biosynthetic reactions. These essential processes generate, as by-products, reactive intermediates, and oxidizing agents, which in turn damage mitochondrial proteins and lipids (10). To this end, distinct mitochondrial quality control mechanisms – the degradation of unfolded mitochondrial proteins by mitochondrial proteases, the elimination of selective cargo by mitochondria-derived vesicles and the elimination of whole mitochondria by mitophagy – function in response to the degree of mitochondrial damage present (10–14).

Nowhere is the requirement for effective mitochondrial quality control systems more important than in neurons, where high energetic demands and need for high calcium-buffering capacity due to action potential-driven calcium influxes rely heavily on proper mitochondrial function (15). This mitochondrial dependence renders neurons especially vulnerable to mitochondrial damage, and, in turn, efficient and properly functioning mitochondrial quality control pathways are paramount to neuronal survival. Highlighting this are genetic studies demonstrating the involvement of genes regulating mitochondrial morphology – MFN2 and GDAP-1 in Charcot-Marie-Tooth type2A, as well as OPA1in Optic Atrophy – and mitochondrial quality control – AFG3L2 in Spinocerebellar Ataxia type 28, parkin and PINK1 in PD – in neurodegenerative disease (16–23).

## The PINK1/Parkin Pathway: A Link between Mitochondrial Quality Control and Parkinson’s Disease

It has been hypothesized recently that the decline in mitochondrial function observed in PD may stem from the rapid deregulation of mitochondrial quality control mechanisms in patients affected by the disease (10, 24). Importantly, recent studies have implicated two genes linked to autosomal-recessive juvenile parkinsonism (AR-JP) in humans – PINK1, a mitochondrially targeted serine/threonine kinase, and parkin, an E3 ubiquitin ligase – in a mitochondrial quality control pathway involving the degradation of damaged mitochondria by autophagy.

Initial genetic evidence from *Drosophila* had suggested that both parkin and PINK1 function in a common pathway regulating mitochondrial morphology by promoting mitochondrial fission – either by inhibiting the pro-fusion protein Fzo1 (the major *Drosophila* mitofusin homolog) or by activating the pro-fission protein Drp1 (25, 26). However, a clear consensus of how these genes affect morphology in mammalian cells has yet to be established (27–29). In regulating mitochondrial function, however, PINK1 has been shown to promote mitochondrial respiration and increase mitochondrial membrane potential (Δψ_m_) (30–32), with a specific link to complex I (33, 34), as well as proper calcium homeostasis in mammalian cell lines (35–38).

Overwhelming evidence in mammalian cell lines has implicated parkin and PINK1 in the mitophagic degradation of dysfunctional, depolarized mitochondria. Upon ablation of Δψ_m_ by the chemical uncoupler CCCP, the Δψ_m_-dependent mitochondrial import of PINK1 – a polypeptide that, basally, is rapidly turned over by proteases once inside mitochondria – is halted, allowing PINK1, bound to the TOM complex, to build up on the outer mitochondrial membrane (39–42). Here, PINK1 recruits parkin from the cytosol, in a manner dependent on functional PINK1 kinase activity, and promotes parkin’s E3 ubiquitin ligase activity, possibly through direct phosphorylation of parkin by PINK1 (43–47). Once recruited to depolarized mitochondria, parkin-dependent ubiquitination and proteasomal degradation of outer membrane proteins – notably the mitofusins, VDACs, and Miro – ultimately lead to autophagy, a step that possibly involves the rupture of the outer mitochondrial membrane (43, 48–55). PINK1-/parkin-dependent mitophagy enlists the canonical ATG (*a*u*t*opha*g*y-related gene) pathway, originally identified in yeast (56). The ubiquitination of mitochondrial proteins by parkin has been suggested to recruit ubiquitin-binding adaptor proteins, such as p62/SQSTM1, to depolarized mitochondria (43, 57, 58). This in turn was shown to induce mitochondrial clustering around the nucleus (43, 57, 58), possibly facilitating the autophagy of mitochondria by increasing their proximity to the endoplasmic reticulum, a possible source of autophagic membranes. Although PINK1/parkin mitophagy has not been fully characterized with respect to the canonical ATG pathway, the requirement for LC3, p62, and ATG5 suggests that depolarization-induced, PINK1-/parkin-dependent mitophagy indeed makes use of the conserved ATG pathway to remove damaged mitochondria.

Clearly, the ability to pharmacologically disrupt Δψ_m_ has enabled the study of the PINK1/parkin pathway using a robust and effective paradigm, although parkin-dependent mitophagy has also been observed under less severe conditions. For example, in fusion-deficient cells, parkin recruitment to depolarized mitochondria (arising from uneven fission) has been demonstrated at the steady-state (50). Furthermore, in cells harboring severe mtDNA mutations, parkin has been shown to selectively remove dysfunctional mitochondria over time (59). However, a truly robust, physiological assay with which to determine the effectiveness of PINK1- and parkin-dependent mitophagy has remained elusive.

## Types of Mitophagy Under Physiological Conditions

**Figure 1:**
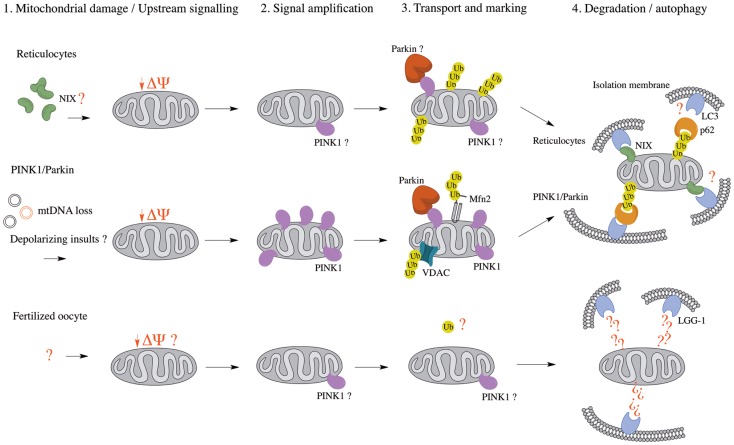
**Comparison of known mitophagy pathways (Reticulocytes, PINK1/Parkin, and Fertilized oocyte) in four major steps**. (1) Upstream signaling or mitochondrial damage activates mitophagy pathways. (2) The initial mitochondrial signaling or mitochondrial damage converges at a flagship protein, which amplifies the signal. (3) Mitochondria are transported to isolation membrane-rich sites and marked for either proteasomal or autophagic degradation. (4) Mitochondria are enveloped by isolation membranes and delivered to autophagosomes. Δψ, mitochondrial membrane potential; LC3, microtubule-associated protein 1 light chain 3; LGG-1, LC3, GABARAP, and GATE16 family 1; Mfn2, mitofusin 2; mtDNA, mitochondrial DNA; NIX, NIP3-like protein X; PINK1, PTEN-induced putative kinase 1; Ub, ubiquitin; VDAC, voltage-dependent anion channel.

Selective mitophagy (depicted in Figure 1) is critical during the development of cells that specifically degrade their mitochondria as they mature. The most-studied example concerns red blood cells (RBCs), which lose their mitochondria in order to transport oxygen instead of consuming it (60). While it was long known that RBCs are devoid of nuclei and organelles such as mitochondria and Golgi apparatus (61), only recently have studies identified mitophagy as the mechanism by which mitochondria are removed (62). Mitophagy in RBCs occurs canonically, according to the conserved ATG protein pathway (56), as well as through a redundant ATG7-independent mechanism involving NIX/BNIP3L, a protein related to Bcl-2 (63–65). Moreover, NIX/BNIP3L has been shown to be an essential mediator of mitochondrial depolarization prior to autophagy (66, 67).

The observation that both NIX- and parkin-/PINK1-dependent mitophagy seem to rely on mitochondrial depolarization as an upstream mechanism prior to autophagy suggests a conserved mechanism among pathways (Table 1), whether they are programed (reticulocyte differentiation) or reactionary (parkin/PINK1). Indeed, NIX was found to be necessary for LC3 activation following Δψ_m_ loss and parkin recruitment to the mitochondria, suggesting it might be essential to the parkin/PINK1 mitophagy pathway (63). This also raises the possibility that parkin and PINK1 function in a programed mitophagy pathway, although it would be unlikely to involve RBCs, as one would then expect parkin- or PINK1-associated PD patients to present with anemia (68).

**Table 1:** **Comparison of mitophagy pathways**.

	Parkin/PINK1	Reticulocytes	Fertilized oocyte
Dependence on ATG family proteins	Yes (LC3, ATG5)	Yes (LC3)	Yes (LGG-1)
Ubiquitination of mitochondria prior to autophagy	Yes	N/A	No
Known, essential pathway components	Parkin, PINK1, HDAC6 (88), Ubiquitin-proteasome system (UPS) (48), VDAC1, 2, and 3 (89), Ambra1 (90), NIX (63)	NIX	N/A
Loss of ΔΨ_m_	Yes	Yes	N/A
Complete loss of mitochondria	Yes	Yes	Yes
Type of mitophagy	Reactionary (?)	Programed	Programed

A second type of programed mitophagy consists of the elimination of paternal mitochondria in the fertilized oocyte (69). While the notion that mitochondrial DNA is inherited uniquely from the mother has been long established, only recently have studies in *C. elegans* found that the degradation of paternal mitochondria, as well as its mtDNA, occurs through mitophagy (70–72). Whereas autophagy of paternal mitochondria was demonstrated to rely on the ATG-associated machinery for mitochondrial degradation, it was also shown that mitochondrial ubiquitination does not appear prior to engulfment, as opposed to mitophagy occurring during reticulocyte maturation and the parkin/PINK1 pathway (Table 1). However, further work on autophagy of paternal mitochondria, clarifying whether mitochondria are depolarized prior to engulfment, remains to be done; it would be interesting to see if parkin and PINK1 are implicated in this pathway, possibly by examining the occurrence of autophagy of paternal mitochondria in fertilized ova of parkin- or PINK1-null mice. Interestingly, parkin- and PINK1-null flies both show mitochondrial abnormalities in embryonic development, characterized by swollen or vacuolated nebenkern – spheres encompassing two giant mitochondria in the developing fly spermatid – which result in male sterility (73–75). These findings suggest that, in spermatogenesis, parkin and PINK1 may have a role in mitophagy-related events.

In essence, it seems that many mitophagic pathways utilize the canonical ATG-associated machinery, yet differ both in the manner through which mitochondria are signaled for autophagosomal engulfment and in upstream signaling mechanisms. In the case of parkin-/PINK1-dependent mitophagy, it is the latter that is poorly defined, and it is plausible that a programed mitophagic pathway, utilizing parkin and PINK1, exists in mammals and contributes to PD pathogenesis. In addition, we have already described that PINK1 can accumulate on mitochondria upon disruption of mitochondrial import in the absence of depolarization (39), suggesting that several upstream mechanisms may impinge on a canonical, programed parkin/PINK1 mitophagic pathway.

## Mitophagy in Parkinson’s Disease

Although studies examining parkin-/PINK1-dependent mitophagy as a quality control mechanism have relied heavily on the use of chemical uncouplers in heterologous cell culture (42, 43, 45, 47, 50), the existence and relevance of such a pathway in neurons has remained elusive, based on a handful of studies relying predominantly on parkin overexpression (76–78). Alarmingly, other groups were unable to show recruitment of overexpressed parkin in neurons following mitochondrial depolarization (79), or showed that endogenous parkin failed to mediate mitophagy in neurons and cultured cells (80).

As described previously, complete autophagy of the mitochondrial network can occur in many cell types in response to intrinsic or extrinsic signals. Neurons, however, cannot switch to glycolytic metabolism (as an ATP-generating mechanism) during acute mitochondrial stress, and hence are utterly dependent on mitochondria for energy production (81, 82). Therefore, it is unlikely that molecular pathways have evolved to remove the whole mitochondrial network following mitochondrial damage in neurons. Moreover, it has been shown that neurons divert glucose away from glycolysis to the pentose phosphate pathway – in order to maintain a high level of reduced glutathione – and instead generate ATP predominantly through OXPHOS (83, 84). As such, disruption of the OXPHOS process by uncouplers or other mitochondrial toxins results in a bioenergetic crisis inherent to neurons, and may contribute to the ambiguity surrounding findings concerning the PINK1/parkin pathway obtained from this cell type.

Thus, two important questions arise from the controversy surrounding the relevance of the PINK1/parkin pathway in neurons: (1) whether or not parkin is recruited to depolarized mitochondria in these cells and, if so, (2) what is the physiological role of this recruitment in neurons? While parkin recruitment was shown to be robust and reproducible in immortalized cells, the data indicate that this is more variable in neurons. This is not so surprising since neuronal culture protocols carry many more variables than those for immortalized cells. When analyzing data from the five studies on parkin-/PINK1-dependent mitophagy in neurons (Table 2), we find that many components of neuronal media could influence parkin translocation. Cai and colleagues used inhibitors of apoptosis in their neuronal culture (the caspase inhibitor Z-VAD-FMK) to counter the effects of high doses of chemical uncouplers triggering apoptosis in an environment devoid of protective glia. While these conditions do lead to parkin translocation in neurons, they may also mask the normal physiological reaction of neurons to gross depolarization of the mitochondrial network. It is unlikely that neurons have evolved to adapt to this type of insult, and apoptosis may be the physiological response. Interestingly, in the study (77) that showed the highest percentage of parkin translocation (about 70%), it was found that recruitment was dependent on the absence of antioxidants (in the form of the B-27 supplement) in the media. Taken together, these factors might explain why some groups (79) were not able to detect significant parkin translocation upon mitochondrial depolarization in neurons. Importantly, parkin translocation was also much slower (12–24 h) in the study that used B-27 than in studies without it (4 h). This indicates that neuronal cultures containing antioxidant supplements may counteract the action of chemical uncouplers. It would however be important to determine if, under growth conditions lacking antioxidants, neurons can survive over long time periods following mitochondrial depolarization. In light of these recent studies, we conclude that parkin can translocate to mitochondria in neurons following depolarization, given the proper culture conditions. However, most of these studies did not quantify mitophagy following parkin translocation (76–78). While Seibler and colleagues found that parkin-positive cells have reduced mitochondrial DNA copy numbers after exposure to CCCP, they did not rule out decreased mitochondrial biogenesis as a possible mechanism. This raises the question of whether parkin translocation proceeds to mitophagy in neurons, or plays another role. Rakovic and colleagues addressed this question by looking at the degradation of a number of mitochondrial proteins both at the outer membrane, the inner membrane, and the matrix. Surprisingly, they found that even when overexpressing parkin in induced pluripotent stem (iPS) cell-derived dopaminergic neurons, parkin does not promote mitophagy upon mitochondria depolarization. However, given that the kinetics of parkin recruitment to mitochondria seem considerably slower in neurons (70% recruitment at 4 h) than in immortalized cells (100% at 2 h) (Table 3), it is plausible that mitophagy may proceed more slowly and may not be detectable at 16 h with the 1 μM valinomycin used by Rakovic and colleagues. Again, one obvious issue may be that incubating neuronal cultures with chemical uncouplers for an extended period may induce apoptosis. Adding apoptotic inhibitors may circumvent this limitation, allowing for the study of parkin-dependent mitophagy in neurons on a longer time scale, as shown by Cai and colleagues. Whereas this allowed them to show colocalization between autophagic markers and mitochondria in isolated events, Cai and colleagues did not quantify them in parkin-overexpressing versus mock-transfected neurons.

**Table 2:** **Comparison of data on Parkin/PINK1-dependent mitophagy in neurons**.

	Cai et al. (76)	Joselin et al. (77)	Seibler et al. (78)	Van Laar et al. (79)	Rakovic et al. (80)
Neuronal type	Cortical	Cortical	IPS-derived dopaminergic neurons	Cortical	IPS-derived dopaminergic neurons
Glial bed	Yes	No	No	No	No
Days *in vitro*	8–10	8	–	9	–
Apoptotic inhibitors	Z-VAD-FMK	No	No	No	No
B-27	Yes	No	–	Yes	Yes
Uncoupling agent + time of exposure	10 μM CCCP 24 h	5 μM CCCP 4 h	1 μM Valinomycin 12 h	10 μM CCCP 6 h	1 μM Valinomycin 12 h
% Cells with parkin recruitment	30%	70%	N/A (increased colocalization)	No CCCP effect 25% basal	N/A
Quantified parkin-dependent mitophagy	No	No	No (reduced mtDNA copy numbers)	N/A	Yes (no parkin-dependent mitophagy)
Endogenous parkin recruitment	Yes	N/A	N/A	No	N/A
PINK1 dependence	N/A	Yes	Yes	N/A	Yes

**Table 3:** **Comparison of parkin recruitment in immortalized cells versus neurons**.

	Immortalized Cells (HeLa, Hek293T, SH-SY5Y, MEFs)	Neurons (primary, IPS-derived)
Mean time of parkin recruitment upon ΔΨ_m_ depolarization (more than 30% cell with parkin on mitochondria)	30 min (50)	4 h (77); 6 h (79); 12 h (78); 24 h (76)
Dependence on PINK1	Yes	Yes
Survival after long-term exposure to chemical uncouplers	Yes	N/A; use of apoptotic inhibitors (76)
Complete removal of mitochondria	Yes	N/A

To overcome the limitations of *in vitro* neuronal cultures, Sterky and colleagues crossed MitoPark mice – which develop progressive parkinsonism and mitochondrial abnormalities stemming from the ablated expression of mitochondrial transcription factor A in dopaminergic neurons (85) – with parkin knockout mice. These mice did not show increased neurodegeneration or accumulation of damaged mitochondria, suggesting that parkin had no role in degrading damaged mitochondria (86). Moreover, they overexpressed parkin in the MitoPark mouse and found that parkin was not recruited to mitochondria at the steady-state. While these data suggest that parkin may not be involved in mitophagy in the brain *in vivo*, it is noteworthy that the mitochondrial defects of MitoPark mice have not been fully characterized, and, as such, the mitochondria of these mice may not be sufficiently depolarized to stabilize PINK1 levels and trigger parkin recruitment. Interestingly, a recent study by Vincow and colleagues demonstrated that parkin null flies exhibit a slower turnover of mitochondrial proteins (87). Moreover, they showed that electron transport chain (ETC) protein turnover is especially affected in both parkin and PINK1 single-null flies, suggesting that, under physiological conditions, parkin and PINK1 might have a specific role in regulating the levels of ETC proteins, as opposed to the complete removal of mitochondria following depolarization in cell lines. In light of these results, it is clear that further studies will be required to test whether parkin promotes mitophagy in neurons and what are its consequences *in vivo*.

## Conclusion

Parkin and PINK1 are the first two PD-associated genes to be implicated in a common genetic pathway. More specifically, the association of parkin and PINK1 in a common mitochondrial quality control pathway has consolidated the hypothesis that mitochondrial defects are central to PD pathogenesis. However, the physiological relevance of such a pathway in neurons requires further investigation. Moreover, neuronal parkin and PINK1 may play roles in mitochondrial homeostasis other than degrading damaged mitochondria, such as regulating ETC protein turnover. Upon review of the few, pioneering studies that have aimed to clarify these questions, we conclude that, although it is robust and implicates many other players subsequent to parkin recruitment, the parkin/PINK1 mitophagy pathway still lacks proper upstream signaling characterization. This is reflected in the inability to find a consensus on the proper conditions with which to study this pathway in a more disease-relevant cell type. We also conclude that, while redistribution of parkin to depolarized mitochondria has now been shown in neurons, the physiological role of such recruitment – specifically, whether or not this proceeds to mitophagy – remains elusive. By understanding the physiological function of parkin and PINK1 in neurons, future studies will undoubtedly reveal key molecular mechanisms underlying neurodegeneration and hence novel therapeutic targets for the treatment of PD.

## Conflict of Interest Statement

The authors declare that the research was conducted in the absence of any commercial or financial relationships that could be construed as a potential conflict of interest.
